# “You Never Get a Second Chance”: First Impressions of Physicians Depend on Their Body Posture and Gender

**DOI:** 10.3389/fpsyg.2022.836157

**Published:** 2022-03-21

**Authors:** Felix C. Grün, Maren Heibges, Viola Westfal, Markus A. Feufel

**Affiliations:** Department of Psychology and Ergonomics (IPA), Division of Ergonomics, Technische Universität Berlin, Berlin, Germany

**Keywords:** doctor-patient interaction, non-verbal behavior, physician – patient relations, gender role, body posture, embodiment, power poses, stereotype

## Abstract

A first impression matters, in particular when encounters are brief as in most doctor-patient interactions. In this study, we investigate how physicians’ body postures impact patients’ first impressions of them and extend previous research by exploring posture effects on the perception of *all* roles of a physician – not just single aspects such as scholarly expertise or empathy. In an online survey, 167 participants ranked photographs of 4 physicians (2 female, 2 male) in 4 postures (2 open, 2 closed). The results show that male physicians were rated more positively when assuming open rather than closed postures with respect to all professional physician roles. Female physicians in open postures were rated similarly positive for items related to medical competence, but they tended to be rated less favorably with respect to social skills (such as the ability to communicate with and relate to the patient). These findings extend what is known about the effects of physicians’ body postures on the first impressions patients form to judge physicians’ medical versus social competencies. We discuss practical implications and the need for more research on interaction effects of body postures and physician gender on first impressions.

## Introduction

How a patient perceives his or her physician influences the extent to which information is shared and whether the doctor-patient communication succeeds to promote patient satisfaction, compliance, and, ultimately, health outcomes (Beck et al., 2002; Ha and Longnecker, 2010). First impressions set the stage for a successful doctor-patient interaction (DPI), in particular when encounters are brief due to time pressure and/or when physician and patient meet only once (e.g., in a pre-operative environment) (Decar et al., 2020).

Recently, empirical research has started to elaborate on how non-verbal behavior related to body postures may impact patients’ first impressions of physicians (Kraft-Todd et al., 2017; Forkin et al., 2019). Specifically, a study by Forkin et al. (2019) has shown that physicians who assume high-power poses (open postures, e.g., with arms on the hips) are more likely perceived to be *competent* than when they assume low-power poses (closed postures, e.g., with arms crossed), independent of their gender. Kraft-Todd et al. (2017) extended perceptions of competence with ratings of soft skills such as physicians’ ability to *empathize*, suggesting that physicians need to be more than just scholarly competent to engage with their patients. Their results have shown that physicians assuming open, patient-facing postures are perceived both more professionally competent *and* more empathic than those assuming closed, more introverted postures.

In this article, we further elaborate on Kraft-Todd et al. (2017) extended perspective on physician roles by investigating the impact of open and closed body postures on the perception of *all* roles of a medical expert – using the roles outlined in the CanMEDS framework (Frank et al., 2015) and also on the roles of the patients in the DPI as defined by the Shared Decision Making (SDM) framework (Elwyn et al., 2012). This extended approach allows us to more fully describe how physicians’ body postures affect patients’ first impressions of the roles they and their physicians assume in the DPI.

In the following, we first present research on the effects of non-verbal, embodied behaviors on the DPI, especially of posture, before outlining the diverse roles of physicians and patients in the DPI and the present research. We then elaborate on the design and the results of our randomized online survey, where participants ranked their perceptions of photographs of female and male physicians in open and closed poses. Finally, conceptual and practical implications will be discussed.

### Embodied Aspects of the Doctor-Patient Interaction (DPI)

The quality of the DPI is not only influenced by the communication of health information, emotions, and preferences on a semantic level (Ha and Longnecker, 2010). Its quality is also related to the – notably less researched (Kraft-Todd et al., 2017) – non-verbal, *embodied* relationship between physician and patient. In addition to research on the effects of physicians’ physical appearance related to clothing (Petrilli et al., 2015), congruence in ethnicity (Ferguson and Candib, 2002), or gender (Roter and Hall, 2015), several studies investigated the role that physicians’ body language plays in person perception in general (Carney et al., 2010; Rennung et al., 2016) and in the DPI in particular (Stepanikova et al., 2012; Schmid Mast and Cousin, 2013). Beck et al. (2002) reported that head nodding, forward-leaning, direct body orientation, uncrossed arms and legs lead to higher patient satisfaction. Kraft-Todd et al.’s (2017) research similarly suggested that patients tend to prefer interactions with physicians assuming open poses, that is, with uncrossed arms and the body openly oriented toward them. Forkin et al. (2019) specifically applied Carney et al.’s (2010) power posture research to the DPI and provided evidence that physicians assuming high-power poses (open postures) were rated more likely to be confident, intelligent, and a leader than when they assumed low-power poses (closed postures). To our knowledge, no research has thus far investigated the effects of body postures on the perception of the whole gamut of physician and patient roles in the DPI as laid out by, for instance, the CanMEDS framework (Frank and Danoff, 2007) and the Shared Decision Making (SDM) framework (Elwyn et al., 2012).

Looking to the clinical realm, there is research showing that the DPI is also influenced by gender, with patients appreciating gender-specific and stereotype-related behavior, such as women using a soft voice (Schmid Mast et al., 2008). Interestingly, Forkin et al. (2019) found no effect of how female versus male doctors were perceived in different poses. Other studies report conflicting results on how exactly gender impacts the DPI. One meta-analysis showed that patients generally prefer interacting with male physicians (Hall et al., 2011), but there are also studies, which indicated that physicians’ gender does not affect how patients rate their satisfaction and confidence in physicians (Solnick et al., 2020) or that patients favor female gynecologists when it comes to a consultation process (Christen et al., 2008). Nonetheless, women, in the health care sector (Burgoon et al., 1991; Schmid Mast, 2004) and beyond (Cuddy et al., 2004), are perceived less benevolently when behaving incongruent to gender stereotypes, while men – to the contrary – are sometimes rewarded for such behavior, for instance, when being perceived as acting in a particularly caring manner (Blanch-Hartigan et al., 2010; Hall et al., 2014, 2015). In summary, the inconsistent findings of gender effects on the perception of physicians warrant further research.

### Physicians’ and Patients’ Roles in the Doctor-Patient Interaction

There is a potential range of skills and associated doctoral roles, from expert know-how and leadership on the one end to social skills and being a good listener on the other end, which might be impacted by different body postures. We turned to the CanMEDs framework (Frank and Danoff, 2007) in our research to ensure that we substantiate the whole gamut of roles a physician might assume in the DPI, using a broad and validated spectrum. The CanMeds framework has been developed for medical education and details six roles, which a physician needs to master to become a medical expert: the Professional, the Communicator, the Scholar, the Health Advocate, the Leader, and the Collaborator (Frank et al., 2015). Albeit developed for medical education, the CanMEDS roles are operationalized and discussed in health care research as well (Ringsted et al., 2006; Dwyer et al., 2014; Prozesky et al., 2019). Based on research, educational principles, and stakeholder consensus, each role has been conceptualized with respect to observable key competencies, which provide an ideal basis for empirical study (Frank and Danoff, 2007).

In addition, we referred to the Shared Decision Making (SDM) framework to identify patient roles to include in our study. According to the SDM framework, patients’ interests are met most effectively if physicians involve them collaboratively in the decision-making process (Elwyn et al., 2012). That is, SDM mandates that not only physicians, but also patients play an active role in the DPI (Charles et al., 1997). Thus, research on the effect of physicians’ body postures on the DPI should also consider any effects on the patient’s perception of their own role. To do so, we considered the basic steps involved in the SDM process, related to the patient’s readiness to ask questions, share preferences with their doctors, weigh pros and cons of a treatment, and, ultimately, come to an informed decision, even if it deviates from the physician’s recommendation (Charles et al., 1999).

### The Present Study

In our research, we extended existing studies, which showed that participants rate physicians’ competence and ability to empathize in open postures more favorably compared to physicians assuming closed postures, independent of physician gender (Kraft-Todd et al., 2017; Forkin et al., 2019). To test the robustness of these findings, we operationalized the independent variables based on the original stimuli used by Forkin et al. (2019) – visuals of two male and two female physicians, each assuming two open and two closed postures – and retained the original outcome variables. We extended the set of outcome variables to explore the effect of physicians’ body postures on (1) the perception of *all* roles of a medical expert as defined by the CanMEDS framework and (2) participants’ perceptions of their own roles in an imagined interaction with these physicians based on the SDM framework.

## Materials and Methods

### Participants

We aimed to recruit about 200 adult participants from the general population in the United States to be able to relate our findings to the study by Forkin et al. (2019), which also used a sample of 200 participants, and to test the robustness of the effect they identified. The data were collected *via* the clickworker marketplace MTurk, which is an established tool to recruit participants in the US (Mortensen and Hughes, 2018).

### Materials

**FIGURE 1:**
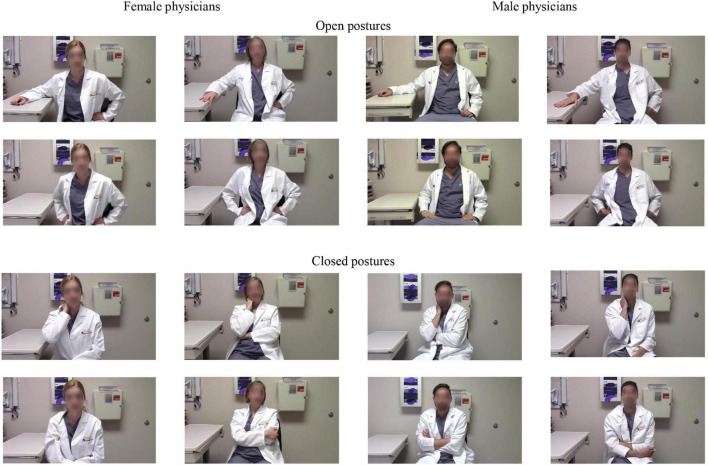
Picture stimuli showing two male and two female physician actors, each in two open and closed poses. The stills were taken from the video stimuli used in the study by Forkin et al. (2019) with the permission of the authors.

Forkin et al.’s (2019) original stimuli consist of videos featuring two female and two male white physician actors, each assuming two open postures (open posture 1: Hands on the hip; open posture 2: One hand on the hip, the other hand on the table) and two closed postures (closed posture 1: Crossed arms; closed posture 2: One arm on the belly and the other hand touching the neck). We created screenshots from the original 2-min videos, featuring each physician actor (2 male and 2 female actors) in each position (2 closed and 2 open postures), making a total of 16 stills (see Figure 1). We decided to use stills in order to focus on the effects of body postures and reduce potentially interfering influences due to gestures, noises, voices, and/or acting abilities. Thus, with these stimuli, we could test the robustness of the posture effects using the same stimuli as Forkin et al. (2019) but in a more controlled presentation format (stills versus videos).

Dependent variables are summarized in Table 1. We first included variables from Forkin et al.’s (2019) original study, which asked participants to rank their confidence in the physicians, the physicians’ intelligence, “How likely you would be to choose the anesthesiologist to care for your family member?” and “Which of these four physicians seems most like a leader?” We also included a control question about how powerful the physicians were perceived based on previous research linking open postures to the concept of power (Forkin et al., 2019).

**TABLE 1:** Overview and abbreviations of the dependent variables, sorted by sources.

Question	Item abbreviation
*Items from the study by* Forkin et al. (2019)	
**Ranking question in the format “Rank each physician in order of…”**	
…their confidence.	Confidence
…how likely you would be to choose the anesthesiologist to care for your family member.	Family Care
…their intelligence.	Intelligence
**Ranking question in the format “Which of these four physicians seems most like a…”**	
…leader.	Leader
*Item connected to power research* (Hall et al., 2005; Carney et al., 2010)	
**Ranking question in the format “Rank each physician in order of…”**	
…their power.	Power
*Items connected to the CanMEDS roles* (Frank et al., 2015)	
**Ranking question in the format “Rank each physician in order of…”**	
…how good they seem at communicating.	Communicator
…how likely they seem to be a team player.	Collaborator
…how much they seem to care for patient needs.	Health Advocate
…their commitment to ethical behavior.	Professional (ethics)
…their commitment to you as a patient.	Professional (patient)
…how likely they make evidence-based decisions.	Scholar
*Items connected to shared decision making* (Elwyn et al., 2012)	
**Ranking question in the format “Rank each physician in order of how comfortable…”**	
…you would feel to ask them questions.	Asking Questions
…you would feel to make a decision that deviates from their recommendation.	Deviating Decisions
…you would feel to tell them about your personal preferences.	Personal Preferences
…you would feel to discuss pros and cons of treatments with them.	Pros and Cons

To extend our research beyond a focus on physicians’ perceived professional competencies, we included questions to cover all six roles of the CanMEDS framework. To do so, we selected items based on the operational definitions of the key competencies that are associated with each CanMEDS role (Frank et al., 2015), reformulating the competencies in laypersons’ terms where needed (e.g., a Health Advocate is a “physician who cares for patient needs”). Finally, we added four ranking questions to assess the effect of physicians’ posture on patients’ perceptions of their own role in the DPI based on the central steps of the SDM process (i.e., the patients perceived abilities to ask the physician questions, reveal their preferences, discuss pros and cons, and make preference-sensitive decisions, even if at odds with the physician’s recommendation) (Charles et al., 1997).

### Design

The 16 pictures of physicians in different postures were assembled in 4 sets of 4 pictures based on an orthogonal Latin Square design, so that each posture and each person was featured once in each set. Participants were randomly assigned to one of these four sets and asked to rank order the pictures with respect to the dependent variables using drag and drop. To answer the question “Which of these four physicians seems most like a leader,” participants were asked to select the one picture they associated most with a leader.

### Procedure

We created the survey with the online survey tool EFS survey by questback. The survey was reviewed and accepted by an ethics committee (review number: MEI_02_20190821). Participation was voluntary, and the participants gave their consent by explicitly clicking a checkbox before they started. A short introductory text asked participants to imagine that they would consult a team of four anesthesiologists before a surgery and were then asked to rate their perceptions of these physicians. The cover story was chosen because before surgery patients and anesthesiologists tend to meet only once and they have no previous relationship so that questions about first impressions seem appropriate. Each rating question was presented on a separate webpage and in a random order. At the end of the survey, participants’ gender, age, education level, ethnicity, and recent physician visits were assessed as covariates. After completing the survey, the participants could redeem a remuneration of $0.50 by sending a code to MTurk. The online survey was published, and the data collection started on September 19, 2019. The survey was closed on September 23, 2019, when the prepaid units for 200 participants were exhausted. Six participants filled the survey but did not redeem the incentive, resulting in a total of 206 raw data sets.

To secure the quality of the data collected *via* the fast-paced MTurk online recruiting tool (Hauser et al., 2018), we installed four measures of quality control to circumvent problematic rating behavior, such as fast and systematic answering. First, to be eligible, participants were required to pass a tutorial with logical ranking questions (*n* = 141 missed this eligibility criterion). Second, during the survey, logical test questions were mixed with the experimental questions, and answering one of them wrong led to exclusion (*n* = 1 was excluded due to this test). Third, each question was shown for a couple of seconds before a participant could answer the ranking question, providing sufficient time to look at the picture. Finally, the participants could invalidate their data without losing remuneration by admitting to having rushed through the survey or not taken the time to read all questions. Of 206 participants who managed to take the survey, 39 invalidated their data using this option, leaving data of 167 participants (81%) to be included in the analysis. This reduction of sample size may affect the statistical power, but it improves the quality of the data.

### Analysis

We chose a cumulative link mixed model (clmm) with random effects to (1) check for “nesting effects” of interdependent data and (2) assess the effects of the independent variables on the ordinal dependent variables. Although we used an orthogonal Latin Square design to create four sets of pictures counterbalancing physician postures and gender, we first checked whether individual pictures or the different sets of pictures showed nesting effects. Also, because every participant ranked four physicians with respect to each dependent variable in a within-subject design, we controlled for nesting effects of individual raters’ answers.

Once interdependent data had been accounted for, we used clmm to calculate odds ratios of physicians’ body posture, gender, and their interactions with respect to the dependent variables. We first ran the full model with gender, posture, their interaction, and all covariates (i.e., age, education, participants’ gender, and ethnicity) included. Then we removed covariates and interaction terms from the model if they were non-significant. For *post hoc* analyses of significant interaction terms and main effects, we calculated the simple effects of posture separately for pictures of female and male physicians. To do so, we repeated the same analysis twice. In a first analysis, we included only the ratings of female physicians to detect the simple effects of open and closed postures. In a second analysis, we ran the same analysis again, including only the ratings of male physicians. The data were computed with RStudio V1.2.5001 and R V. 3.6.1. Plots were generated with GGplot.

## Results

### Descriptive Statistics

As Table 2 shows, slightly more than half of the 167 participants with valid data sets were male (57.5%; *n* = 96), about 42% were female (*n* = 70). Most participants described their ethnicity as White (57.5%; *n* = 96), followed by Asian (30.5%; *n* = 51) and Black/African American (6.6%; *n* = 11). Participants’ age ranged from 20 to 70 years, with a mean age of 35 years and a standard deviation of about 11 years. About 76.0% of the participants had a bachelor’s degree or higher education. On average, the participants saw a doctor about thrice (Mean: 3.2; range: 0–12) in the last 12 months. The average completion time was 12 min and 53 s.

**TABLE 2:** Participants’ demographics.

	*N*	%
**Gender**		
Male	96	57.5%
Female	70	41.9%
Diverse	1	0.6%
**Total**	**167**	**100%**
**Ethnicity**		
White	96	57.5%
Black/African American	11	6.6%
Hispanic/Latino	7	4.2%
Asian	51	30.5%
Other	2	1.2%
**Total**	**167**	**100%**
**Education**		
Less than high school	2	1.2%
High school	38	22.8%
Bachelor’s degree	107	64.1%
Master’s degree	19	11.4%
PhD	1	0.6%
**Total**	**167**	**100%**

### Check for Nesting Effects and Main Results

We first tested for nesting effects and found that neither the four answers provided by each participant nor the four sets of four photographs nor the individual photographs systematically influenced the results. In a second step and with both independent variables and their interaction included, we tested for significant effects of covariates. For all dependent variables, the covariates (i.e., participants’ gender, their age, education level, ethnicity, and the frequency of their recent doctor visits) had non-significant effects and were thus excluded from the models. Thirdly, we excluded non-significant interactions terms. The results are summarized in Table 3.

**TABLE 3:** Results of logistic functions, regressing physicians’ posture, gender and posture × gender on the dependent variables.

Dependent variables	Physicians’ gender_1_		CI 90%		Physicians’ body posture_2_		CI 90%		Interaction (Gender × Body posture)		CI 90%	
	Sig.	OR	LL	UL	Sig.	OR	LL	UL	Sig.	OR	LL	UL
***Items from Forkin et al. (2019)***												
Confidence	*p* < 0.001***	0.49	0.35	0.68	*p* < 0.001***	2.22	1.61	3.08	0.015*	1.98	1.25	3.14
Family Care	*p* < 0.001***	0.30	0.21	0.41	0.077	0.71	0.51	0.98	*p* < 0.001***	4.70	2.95	7.50
Intelligence	*p* < 0.001***	0.37	0.27	0.52	0.476	1.15	0.84	1.58	*p* < 0.001***	2.69	1.70	4.28
Leader	0.035*	0.68	0.50	0.92	*p* < 0.001***	3.09	2.26	4.25	ns			
**Power related item**												
Power	0.104	0.80	0.63	1.00	*p* < 0.001***	2.59	2.05	3.28	ns			
***CanMEDS related items***												
Communicator	*p* < 0.001***	0.42	0.30	0.59	0.682	0.92	0.67	1.28	*p* < 0.001***	6.00	3.76	9.62
Collaborator	0.044*	0.67	0.49	0.93	0.072	0.70	0.51	0.97	*p* < 0.001***	3.89	2.45	6.19
Health Advocate	*p* < 0.001***	0.43	0.31	0.60	0.014*	0.62	0.44	0.85	*p* < 0.001***	4.58	2.88	7.29
Professional (ethics)	*p* < 0.001***	0.38	0.28	0.54	0.540	0.89	0.64	1.23	0.010**	2.05	1.30	3.25
Professional (patient)	*p* < 0.001***	0.34	0.24	0.47	0.064	0.69	0.50	0.96	*p* < 0.001***	4.59	2.88	7.32
Scholar	0.002**	0.64	0.52	0.82	0.001***	1.62	1.29	2.04	ns			
**Shared Decision-Making related items**												
Asking Questions	*p* < 0.001***	0.50	0.36	0.70	0.064	0.69	0.50	0.96	*p* < 0.001***	4.38	2.76	6.98
Deviating Decisions	0.825	0.96	0.70	1.32	0.012*	0.61	0.44	0.84	0.009**	2.08	1.32	3.29
Personal Preferences	*p* < 0.001***	0.41	0.30	0.58	0.051	0.68	0.49	0.94	*p* < 0.001***	5.23	3.29	8.35
Pros and Cons	*p* < 0.001***	0.48	0.35	0.66	0.090	0.71	0.51	0.99	*p* < 0.001***	4.52	2.85	7.20

*p < 0.05, **p < 0.01, ***p < 0.001. ns, non-significant. CI = confidence interval, LL = lower limit, UL = upper limit. _1_, male physicians compared to female physicians. _2_, open postures compared to closed postures.

### *Post hoc* Simple Effects Analyses

We now report the results of the *post hoc* simple effects analyses for dependent variables with significant interaction terms and for main effects in all other cases. For male physicians, simple effects of body posture were significant for all dependent variables, except for the variable Deviating Decisions, and showed identical patterns. That is, male physicians occupying open postures are persistently ranked higher than those in closed postures (see Table 4).

**TABLE 4:** Simple effects *post hoc* analyses, regressing physicians’ posture on the dependent variables, separately for pictures of female and male physicians.

	Female		Male	
Group 1	Estimate	*p*-value	Estimate	*p*-value
Confidence	0.415	*p* < 0.001***	0.711	*p* < 0.001***
Intelligence	0.073	0.456	0.528	*p* < 0.001***
Leader	0.406	*p* = 0.001**	0.780	*p* < 0.001***
Power	0.400	*p* < 0.001***	0.550	*p* < 0.001***
Scholar	0.144	0.143	0.338	*p* < 0.001***
**Group 2**				
Family Care	−0.173	0.079	0.579	*p* < 0.001***
Communicator	−0.040	0.686	0.876	*p* < 0.001***
Collaborator	−0.178	0.070	0.494	*p* < 0.001***
Health Advocate	−0.234	0.018*	0.540	*p* < 0.001***
Professional (ethics)	−0.060	0.540	0.300	0.003**
Professional (patient)	−0.174	0.077	0.603	*p* < 0.001***
Asking Questions	−0.177	0.072	0.604	*p* < 0.001***
Deviating Decisions	−0.235	0.018*	0.125	0.205
Personal Preferences	−0.189	0.055	0.664	*p* < 0.001***
Pros and Cons	−0.162	0.100	0.619	*p* < 0.001***

*p < 0.05, **p < 0.01, ***p < 0.001. Estimate = beta coefficient, open postures compared to closed postures.

In contrast to Forkin et al.’s (2019) study, which did not show any significant effects of gender, we found that there were differences in how female physicians were rated. Two major patterns emerged, splitting the dependent variables into two groups for women: In a first group of dependent variables, female physicians were rated as their male counterparts, that is higher in open compared to closed postures (see Group 1 in Table 4). In this first group, the simple effects are positive and, except for the items Intelligence and Scholar, significant. In a second group of dependent variables, ratings of female physicians showed a reversed trend, that is female physicians in open postures tended to be rated *lower* compared to female physicians in closed posture (see Group 2 in Table 4). The simple effects in the latter group are all negative and approach significance in most cases, but only the items Health Advocate and Deviating Decisions reach significance at the 0.05 level.

**FIGURE 2:**
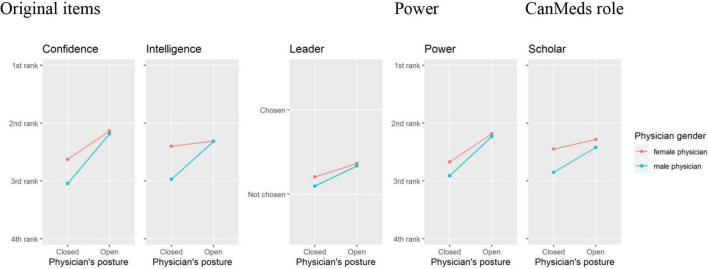
Items for which open postures have a similar trend for male and female physicians.

The pattern showing a similar rating of female and male physicians can be seen in Figure 2. All dependent variables of this pattern have in common that female and male physicians alike tended to be ranked higher in open postures compared to closed postures, and females generally received higher rankings than males.

The pattern, as seen in Figure 2, includes the original items tested by Forkin et al. (2019) related to physician confidence, intelligence, and leadership quality. Confidence and Intelligence show significant interactions of posture and gender. The simple effects reveal that ratings of Confidence were significantly higher for open than for closed postures in both female and male physicians, whereas ratings of Intelligence only increased significantly for male physicians (see Table 4). Female physicians were generally rated higher on Intelligence, independent of posture. For the item Leader, there were significant main effects of gender (OR = 0.68; *p* = 0.035) and posture (OR = 3.09; *p* < 0.001). Specifically, physicians in an open posture were more than three times as likely selected as a leader compared to physicians in a closed posture, and male physicians were less likely selected as a leader than female physicians. Also, the control item assessing perceptions of Power is part of this first rating pattern within which open postures were preferred. Physicians with open postures were about 2.5 times more likely to be rated as powerful than physicians in closed postures (see Figure 2). Finally, a similar pattern with significant main effects of gender (OR = 0.64, *p* = 0.0002) and posture (OR = 1.62; *p* = 0.0001) (see Figure 2) is found with respect to the CanMEDS role Scholar. Again, female gender and open postures increase the likelihood that physicians are considered a scholar.

**FIGURE 3:**
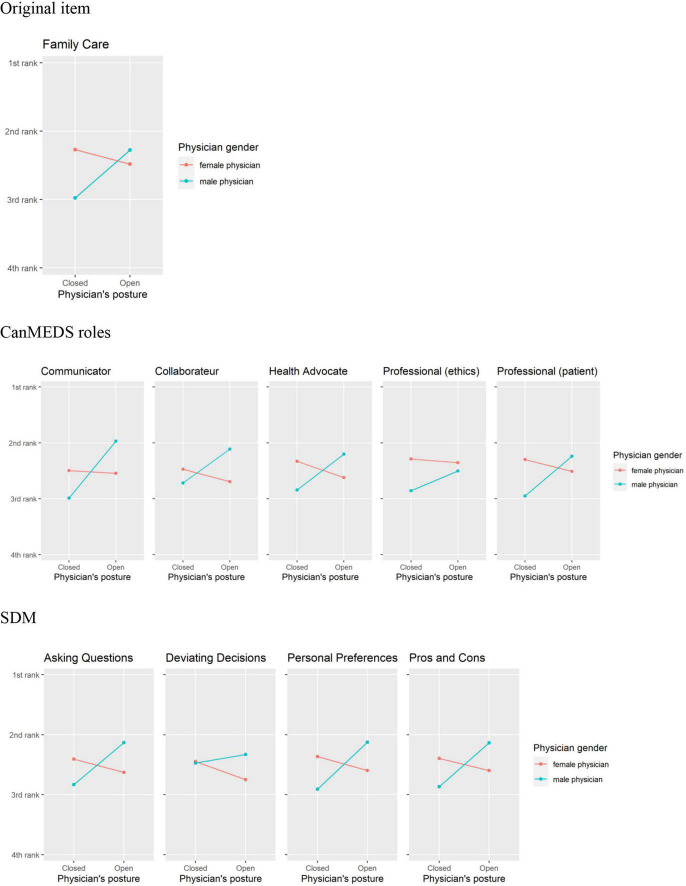
Interaction overview of items, for which open and closed postures have a trend for an X-shaped Interaction for male and female physicians.

For the 10 remaining items (see Figure 3), the interaction terms between posture and gender were significant, showing a pattern which was reversed for female physicians. In other words, for the 10 remaining items male physicians in open postures tended to be ranked higher than those in closed postures, whereas female physicians were rated higher in closed postures than in open postures.

## Discussion

How do body postures shape first impressions of physicians? To answer this question, we set out to replicate previous findings on how patients perceive male and female physicians in different postures and to extend these findings by investigating posture effects on the perception of *all* roles of a physician and the roles of the patient in the DPI. Based on these insights we hope to provide a more nuanced perspective on posture effects than was possible in previous research.

We have found that *male physicians* tend to be perceived as professionally more competent when they assume open body postures. This finding is consistent with Forkin et al.’s (2019) study. In addition to confirming this result, we found that male physicians in open postures are perceived more favorably with respect to *all* roles and competencies defined by the CanMEDS framework. Male doctors in open poses also seem to encourage patients to assume an active role in the DPI, given the more favorable ratings for items measuring patient participation (for example the patients likelihood to ask questions).

For *female doctors* we found a more nuanced pattern. Consistent with Forkin et al.’s (2019) study, female physicians assuming open postures were perceived as professionally more *competent* than those in closed postures. This tendency reversed, however, for items operationalizing CanMEDS roles related to being a communicator, collaborator, or health advocate and for items operationalizing the patients’ roles in the SDM process. That is, unlike their male counterparts, female physicians tended to be rated more positively in closed postures on items related to *social competencies*.

These results relate to but also contrast with prior work by Kraft-Todd et al. (2017). On the one hand, the authors used Fiske et al.’s (2002) Stereotype Content Model (SCM) to assess how physicians in different body postures are perceived on two widely researched dimensions of person perception: perceived “warmth” and perceived “competence” (see also Cuddy et al., 2009). Although they were derived from different theoretical sources, these dimensions map nicely on the pattern in our dependent measures (see Figure 2 versus Figure 3). First, there are variables referring to professional competence and scholarly expertise (Confidence, Intelligence, Scholar, Leader, and Power), and for these variables women in open postures are perceived as more competent. Hence, in their role as a *Competent Professional*, as we would caption the construct underlying this group of variables, female physicians profit from assuming open postures. The remaining dependent variables associated with the CanMEDS roles of the Communicator, the Collaborator, the Health Advocate and the Professional show the same pattern as the dependent variables assessing the patients’ perception of their own role in the SDM process (such as Asking Questions, Deviating Decisions, Personal Preferences, and Pros and Cons). All of them are associated with communicative and interpersonal skills. With respect to these social skills, female physicians tended to be rated more positively in *closed* postures. Hence, female physicians are more likely to be seen as a *Social Supporter –* as we propose to call the construct underlying this second group of variables – when assuming a closed pose.

On the other hand, Kraft-Todd et al. (2017) did not measure gender differences. Based on an experimental manipulation of females and males in empathic (open) versus unempathic (closed) body postures, they reported that physicians in open, empathic postures generally tend to be perceived as both competent *and* warm. We found a similar pattern for *male* physicians, although our results provide a more differentiated perspective on the perception of *female* physicians. SCM research, which informed Kraft-Todd et al.’s (2017) work, showed that women in the workforce tend to be rated high in Competence, but low in Warmth, compared to working men, who tend to have high ratings in *both* Competence and Warmth (Cuddy et al., 2004). There is a debate whether this is a *general* pattern of “stereotype backlash” (Rennung et al., 2016), which we cannot resolve with our study. But our and other data (e.g., Schmid Mast, 2004; Schmid Mast et al., 2010) seem to suggest that in this *particular professional context* – the medical domain – female physicians may be penalized as “cold” if they come across as particularly competent by assuming open poses. Future research drawing on the SCM literature is needed to follow up on this *post hoc* explanation of our findings.

### Limitations

We used a within-subjects design asking our participants to rank four physicians who differed systematically with respect to a dependent variable. This design deviates from the natural setup, where patients encounter one physician at a time. Although we used it to replicate and be able to build on previous studies, this design may have artificially highlighted the differences in gender and body posture between the physicians and inflated the observed effects. The robustness of our findings needs to be tested with a between-subjects design in future research.

With respect to the stimulus material, we used a set of 16 static screenshots from video stimuli used in Forkin et al.’s (2019) study to be able to build on and extend their findings. On the one hand, the screenshots omitted the vivid impression and non-verbal cues of the videos [e.g., related to the tone of the voice or the frequency of nods and smiles (Beck et al., 2002)]. On the other hand, the stills reduced potential confounding effects related to physicians’ voice and gestures and helped to focus our study on the main relationships of interest – the link between body postures, gender, and the first impression that is formed of a physician. In this sense, static screenshots represent face-valid stimuli for accumulating evidence on how first impressions are formed based on body postures in high-paced environments such as the pre-operative environments (Decar et al., 2020). Also, in times of rising demand for telehealth, the computer-based setup (instead of a face-to-face setting) might have added an element of ecological validity. Nonetheless, future research should validate our results in more realistic settings and with more realistic stimuli, for instance, by first observing embodied interactions between patients and physicians and their typical postures in a clinical setting and then operationalizing and testing them in realistic face-to-face *and* video consultation settings.

## Conclusion

The results of the present study substantiate previous findings about how body postures shape patients’ first impression of their physicians. We were able to extend prior research by focusing on the effect of postures on patients’ perceptions of *all* facets of a medical expert (based on the CanMEDS roles) and their own role in the DPI (based on the SDM process). Specifically, male physicians assuming open postures were perceived more favorably throughout. Conceptually, this finding adds to the existing evidence by widening the observed range of posture effects beyond perceptions of professional competence. Female physicians were more likely seen as a Competent Professional when assuming an open posture but less so as a Social Supporter. This finding provides initial evidence that posture effects might be prone to gender-related stereotypes. We suggest the Stereotype Content Model as a productive framework to further explore the effect of stereotypes on physician perception.

### Practice Implications

Body postures influence patients’ perceptions. Thus, in addition to training verbal aspects of the doctor-patient interaction, medical professionals, educators, and students should be made aware of the non-verbal, embodied dimensions of the DPI, in particular how their own posture may influence the first impression they make on their patients. But rather than introducing “empathic non-verbal training in medical education” for all [as suggested by Kraft-Todd (2017, p. 10)], we believe based on the available evidence that raising awareness on how body postures influence the DPI in light of prevailing gender stereotypes might be more effective to help physicians navigate challenging counseling situations. Heightened awareness might also help to alleviate some of the stereotypes in the long run through consistently addressing and challenging them in the DPI. In the meantime, educators and medical professionals should be aware that for women in the medical domain adopting power poses might come at a cost.

## Data Availability Statement

The data presented in this study are publicly available and can be found in the online repository DepositOnce: http://dx.doi.org/10.14279/depositonce-12355.

## Ethics Statement

This study involved human participants and was reviewed and approved by the Ethics Committee (EK) of the Department of Psychology and Ergonomics (IPA) at Technische Universität Berlin. All participants provided their written informed consent to participate in this study.

## Author Contributions

FG and MF contributed to conception and design of the study. FG organized the database and performed the statistical analysis. FG, MH, and MF contributed to the interpretation of the data. FG and MH wrote the first draft of the manuscript. FG, MH, VW, and MF wrote sections of the manuscript. MH and MF critically revised the manuscript for important intellectual content. All authors read and approved the submitted version.

## Conflict of Interest

The authors declare that the research was conducted in the absence of any commercial or financial relationships that could be construed as a potential conflict of interest.

## Publisher’s Note

All claims expressed in this article are solely those of the authors and do not necessarily represent those of their affiliated organizations, or those of the publisher, the editors and the reviewers. Any product that may be evaluated in this article, or claim that may be made by its manufacturer, is not guaranteed or endorsed by the publisher.
